# Internet-Based Cognitive Behavioral Therapy for Panic Disorder Among Arabic-Speaking Patients: Case Series

**DOI:** 10.2196/83415

**Published:** 2026-11-12

**Authors:** Asala Halaj, Lama Kaiyal, Gerhard Andersson, Jonathan D Huppert

**Affiliations:** 1 Department of Community Mental Health, University of Haifa, 199 Abba Khoushy Avenue, Mount Carmel, Haifa, 3103301, Israel, 972 505554291; 2 Department of Psychology, The Hebrew University of Jerusalem, Jerusalem, Israel; 3 Department of Behavioural Sciences and Learning, Linköping University, Linköping, Sweden; 4 HEI-Lab: Digital Human-Environment Interaction Labs, Lusófona University, Lisbon, Portugal; 5 Department of Health, Education and Technology, Luleå University of Technology, Luleå, Sweden

**Keywords:** internet-based cognitive behavior therapy, panic disorder, anxiety sensitivity, agoraphobic avoidance, Arabic-speaking patients, cultural adaptation, case series

## Abstract

**Background:**

Internet-based cognitive behavioral therapy (ICBT) has shown promise for the treatment of panic disorder (PD), but little is known about its feasibility and use across cultures and languages.

**Objective:**

This study examines the feasibility and preliminary outcomes of an Arabic-translated ICBT program for PD and describes how patients experienced the program.

**Methods:**

Six Arabic-speaking women (aged 21‐50 years, mean 35, SD 10 years) with a primary diagnosis of PD underwent a 16-week guided ICBT program using an Arabic-translated protocol. PD symptoms (Panic Disorder Severity Scale–Self-Report [PDSS-SR]), anxiety sensitivity (Anxiety Sensitivity Index-3 [ASI-3]), and agoraphobic avoidance (Mobility Inventory [MI]) were assessed pretreatment, posttreatment, and 3 months later. The outcomes were analyzed descriptively. Four patients also participated in individual, semistructured posttreatment interviews about their experience of the program, which are reported descriptively as the patient-perspective component of the case series.

**Results:**

The program was completed by 4 of the 6 (67%) patients. Among completers, PDSS-SR scores decreased from 11‐18 pretreatment to 0‐7 posttreatment, ASI-3 scores decreased from 18‐59 to 0‐4, and MI scores decreased from 1.44‐3.82 to 0.00‐1.60, indicating reduced agoraphobic avoidance. Follow-up data were available for 3 of the 4 (75%) completers: 2 maintained their gains, and 1 showed a partial return of symptoms. The fourth reported a recurrence of symptoms posttreatment but could not be reached for follow-up. During the interviews, all 4 patients expressed positive views of the program.

**Conclusions:**

Arabic-translated ICBT appeared feasible and was associated with reductions in panic symptoms and anxiety sensitivity among Arabic-speaking patients with mild-to-moderate PD. These preliminary findings support further investigation with larger samples.

## Introduction

Panic disorder (PD) is a prevalent anxiety disorder characterized by recurrent, intense panic attacks. Common symptoms include rapid heartbeat, sweating, shortness of breath, and dizziness [1]. PD is marked by excessive distress and persistent worry about future attacks. Individuals with PD often avoid situations that may trigger attacks, potentially leading to agoraphobia if this avoidance becomes chronic. A key factor in the development and maintenance of PD is anxiety sensitivity (AS). AS, often referred to as “fear of fear,” is an anxiety-related trait characterized by the fear of anxiety-related physiological sensations. Individuals with high AS tend to catastrophize bodily sensations, believing they lead to negative consequences [2]. AS has been shown to predict the development of various anxiety disorders, especially PD [3]. Given the central role of AS in PD, effective treatments often target this trait.

Cognitive-behavioral therapy (CBT) has emerged as an effective treatment for various anxiety disorders, including PD [4]. CBT has proven effective in reducing AS [5], with reductions in AS often preceding improvements in PD symptoms [6]. Specific CBT protocols have been developed for PD, making it the first-line intervention for this disorder [7]. However, access to traditional face-to-face CBT remains limited for many patients by barriers such as cost, geographical distance, long wait times, and a shortage of adequately trained therapists [8]. To address the substantial discomfort and impairment associated with PD and overcome barriers to traditional therapy (eg, high costs, travel distances, and limited clinician time), internet-based cognitive behavioral therapy (ICBT) has become a viable alternative to face-to-face CBT [9]. ICBT delivers interventions via online platforms that include assessment instruments, treatment materials, and homework assignments [10]. Guided self-help interventions involve clinician interaction for support, tracking, and feedback, enhancing the treatment experience. The efficacy of ICBT has been well-documented in recent years. Research suggests that ICBT is effective for PD with or without agoraphobia, with sustained effects and similar rates of symptom reduction [11]. Additionally, research suggests that ICBT is as effective as face-to-face CBT in reducing symptoms of PD [12]. Over the past 2 decades, global implementation of ICBT has shown clinical improvement, low rates of symptom worsening, and high patient satisfaction. The first ICBT study for PD in Israel [13] found it to be as effective as face-to-face CBT, with improvements maintained for up to 12 months. Moreover, therapist time was reduced by two-thirds, highlighting ICBT’s cost-effectiveness. These promising results have raised interest in applying ICBT to diverse populations, including the Arab population.

A meta-analysis of CBT and ICBT among Arab adults demonstrated effectiveness in reducing anxiety, depression, and posttraumatic stress disorder symptoms [14,15]. However, there is a notable lack of evidence-based ICBT guidelines and disorder-specific systematic reviews tailored to Arab populations, particularly for PD. This gap is especially concerning given the burden of anxiety disorders in the Middle East and North Africa region [16]. Although large-scale epidemiological studies specifically examining PD prevalence in Arab populations remain scarce, recent evidence from Saudi Arabia found that 13.1% of adults met the criteria for PD [17], suggesting that PD may be a meaningful yet underrecognized clinical concern in some Arab populations. The limited cross-cultural evidence that does exist suggests that the expression of panic symptoms may vary across cultures. In a study of patients with PD in Australia, those of Arabic background reported greater fear of dying and a stronger somatic emphasis, whereas those of Australian background reported greater social concerns, such as fear of sweating or losing control [18]. Because that comparison involved a single host culture, it cannot be taken as evidence of a general difference between Arab and Western populations, and further cross-cultural work is needed. However, culturally adapted psychological interventions have proven both acceptable and effective for Arab populations across diverse clinical contexts [14,15], suggesting that adaptation of content, examples, and delivery format is likely to be important for engagement. Nevertheless, to our knowledge, no Arabic-language ICBT protocol for PD has yet been described or evaluated.

To address these gaps, the current case series aims to (1) examine the feasibility and preliminary outcomes of ICBT for Arabic-speaking patients with PD in Israel and (2) describe, based on posttreatment interviews, how patients experienced the Arabic-language program and what they identified as helping or hindering their engagement with it. We hypothesized that panic symptoms and AS scores would decrease from pretreatment to posttreatment, with these improvements maintained at the 3-month follow-up. The second aim is descriptive, and no hypotheses were specified.

## Methods

### Study Design

This study used a prospective staggered baseline case-series design. Six Arabic-speaking patients with a primary diagnosis of PD were randomly and equally allocated to an immediate-start group, which began treatment at the end of recruitment, and a delayed-start group, which began treatment 1 month later. During that month, the delayed-start group completed the same self-report measures as the immediate-start group, with 1 to 2 assessments recorded per patient, providing a period of pretreatment observation. All 6 patients then received the same 16-week guided ICBT program using an Arabic-translated protocol delivered by a single trained therapist. Self-report outcomes were collected pretreatment, posttreatment, and 3 months later (follow-up), as well as weekly during the program; clinician-administered assessments were conducted at pretreatment, posttreatment, and follow-up. Individual semistructured interviews were conducted posttreatment to explore patients’ experiences of the program. Given the small sample size and study design, all quantitative outcomes were analyzed descriptively at both the individual and group levels. Patients were recruited in December 2022. The immediate-start group began treatment in January 2023, and the delayed-start group began treatment in February 2023; patients completed treatment between June and September 2023, and data collection, including the follow-up assessments, was completed in November 2023.

### Participants and Recruitment

We recruited 6 Arabic-speaking patients with PD using social media (eg, Facebook, Instagram, and WhatsApp) and public advertisements. Study inclusion criteria included (1) a primary diagnosis of PD with or without agoraphobia; (2) age 18 years or older; (3) PD duration of at least 2 months; (4) no active suicide risk; (5) absence of current or a history of psychosis or uncontrolled bipolar I disorder; (6) no current psychotherapy for treating PD or history of a complete course of CBT for PD; (7) a stable medication dosage for 3 months; and (8) willingness and ability to use the internet and to read and write in Arabic. All communications with participants were in Arabic.

### Procedures

Interested participants registered on the study website and completed initial symptom screening questionnaires. Eligible candidates were then screened by telephone, and those who passed were invited for a pretreatment clinical interview via Zoom. During this interview, patients were assessed using the Diagnostic Interview for Anxiety, Mood, and Obsessive-Compulsive and Related Neuropsychiatric Disorders (DIAMOND) [19] and the Panic Disorder Severity Scale–Interviewer Edition (PDSS-IE) [20] to confirm PD as the primary diagnosis and to assess PD symptom severity. All patients completed self-report measures of panic symptoms (Panic Disorder Severity Scale–Self-Report [PDSS-SR]) [21], agoraphobic avoidance (Mobility Inventory [MI]) [22], and AS (Anxiety Sensitivity Index-3 [ASI-3]) [23] pretreatment, posttreatment, and 3 months later, as well as weekly during the program. Posttreatment assessments mirrored the pretreatment assessments and were conducted via Zoom. The 3-month follow-up assessments were conducted by telephone, during which the PDSS-IE was administered; patients self-completed the PDSS-SR, ASI-3, and MI online.

### Ethical Considerations

This study was approved by the Social Sciences Ethics Committee of the Hebrew University of Jerusalem (approval number 2022‐07202). All participants gave their informed consent before enrollment by completing an online consent form when registering on the study website and provided additional consent during the pretreatment Zoom interview. All data were deidentified prior to analysis: participants were assigned unique numeric identifiers, and identifying information (eg, names and contact details) was stored separately from the study data on a password-protected server accessible only to the research team. Participants did not receive financial or other compensation for their participation.

### Treatment

Patients were treated via an ICBT version for PD using the same treatment protocol from the study by Strauss et al [13], which was translated into Arabic by 2 researchers from the research team who were native Arabic speakers. One translated the materials, and the second reviewed the translation for accuracy and clarity. Adaptations were made to fit the Arab cultural context. For instance, exposure scenarios involving dating or drinking alcoholic beverages with friends were replaced with situations that are common and comparably anxiety-provoking in the local Arab context, such as visiting family and spending time with friends. The language was also adjusted from formal written Arabic to a clearer, more readable style because formal Arabic can be more difficult to follow in everyday reading. The treatment was administered over 16 weeks, taking individual and clinical needs into consideration. The treatment program consisted of 6 modules, including reading texts, worksheets, videos, and assignments. The modules contained (1) an introduction to the treatment program, (2) psychoeducation, (3) cognitive restructuring, (4) interoceptive exposure, (5) in vivo and interoceptive exposure, and (6) a summary and relapse prevention.

The treatment was administered by a master’s-level psychology student trained in CBT for PD. The therapist received weekly supervision from a senior clinical psychologist to ensure treatment fidelity and address any clinical challenges. The therapist was a native speaker of Arabic and familiar with the cultural context of the participants. Therapist-patient contact occurred in 2 ways. First, after each module, the therapist reviewed the patient’s worksheets and provided written feedback through the platform’s secure messaging system, after which the next module was activated. Second, the therapist initiated contact when a patient had not logged in or progressed for some time, had not completed a weekly questionnaire, or had raised a question. This was usually by telephone call, followed by a platform message if the call was not answered, and occasionally by a brief WhatsApp (Meta Platforms, Inc) or email message. Patients could also message the therapist through the platform at any time. Completers were defined as patients who finished at least the fifth module of the treatment program.

### Measures

All measures were either previously validated in Arabic or translated for this study by native Arabic speakers using translation and back-translation procedures to ensure semantic accuracy. Minor linguistic adjustments were made to enhance cultural and contextual appropriateness for Arabic-speaking patients.

The PDSS-SR [21] is a 7-item self-report measure assessing PD symptom severity, including panic attack frequency, distress, fears and avoidance related to agoraphobia and bodily sensations, and impairment in work and social functioning. Each item is rated on a 5-point Likert scale (0‐4), yielding a total score ranging from 0 to 28, with higher scores indicating greater PD severity. A score of 7 or higher is commonly used as a threshold for clinically significant PD symptoms. The PDSS-SR has demonstrated good internal consistency (Cronbach α=0.92) [21].

PDSS-IE [20] is a clinician-administered measure consisting of 7 items assessing PD symptom severity over the past week across the same domains as the PDSS-SR. Items are rated on a 5-point Likert scale (0‐4), yielding total scores ranging from 0 to 28, with higher scores indicating greater PD severity. A score of 7 or higher is commonly used as a threshold for clinically significant PD symptoms. The PDSS-IE has demonstrated good internal consistency (Cronbach α=0.88) [20].

The ASI-3 [23] is an 18-item self-report measure of AS, defined as the fear of anxiety-related sensations. It comprises three 6-item subscales assessing physical, cognitive, and social concerns. Items are rated on a 5-point Likert scale (0=very little to 4=very much), yielding a total score ranging from 0 to 72, with higher scores indicating greater AS. The ASI-3 has demonstrated good internal consistency for both the total scale and its subscales (Cronbach α range=0.73-0.91) [23]. Scores of 23 or higher are commonly used as an indicator of elevated AS.

The MI [22] is a 27-item self-report measure assessing agoraphobic avoidance across a range of situations. Each situation is rated twice on a 5-point Likert scale (1=“never avoid” to 5=“always avoid”): once for avoidance when alone and once for avoidance when accompanied, yielding 2 mean scores. Higher scores indicate greater avoidance. The MI has demonstrated excellent internal consistency (Cronbach α>0.90) [22]. A mean MI score of 1.5 or higher is often considered indicative of clinically significant avoidance.

The DIAMOND [19] is a semistructured clinician-administered interview designed to assess *DSM-5* (*Diagnostic and Statistical Manual of Mental Disorders, Fifth Edition*) psychiatric disorders, with particular emphasis on anxiety, mood, obsessive-compulsive, and related conditions. It evaluates primary and comorbid diagnoses using standardized diagnostic criteria and has demonstrated good reliability. In the current study, the DIAMOND was used during pretreatment assessment to confirm PD as the primary diagnosis and assess comorbidities.

### Posttreatment Interviews

At the end of treatment, all patients were invited to participate in an individual semistructured interview about their experience with the program. Four patients agreed to be interviewed: 3 completers were interviewed shortly after the posttreatment assessment, and 1 noncompleter was interviewed after withdrawing. Of the remaining 2 patients, 1 stopped responding to the therapist following a recurrence of symptoms posttreatment, and 1 asked for their interview to be postponed and then did not attend 2 scheduled appointments. The interviews were conducted in Arabic via Zoom by the treating therapist (LK), a graduate student pursuing a master’s in clinical psychology and trained in CBT for PD, who was known to all patients and served as their therapist throughout the program. The interviews lasted approximately 60 minutes and were not audio-recorded; the therapist documented each interview in written notes taken during and immediately after it. The interview covered overall satisfaction with the program and whether it had met the patient’s expectations; the perceived helpfulness and difficulty of the individual treatment modules; the experience of the therapeutic relationship and the platform’s messaging system; suggestions for improving the program; and, for patients who discontinued the program, what might have enabled them to complete it. Nondirective follow-up prompts were used to clarify and expand on patients’ responses.

### Data Analysis

Quantitative outcomes and interview material were handled separately as described below. Given the small sample size and case-series design, all quantitative data were analyzed descriptively without inferential statistical testing. We computed individual patient scores and group-level descriptive statistics (means and ranges) for the PDSS-SR, ASI-3, and MI at pretreatment, posttreatment, and 3-month follow-up, as well as for weekly assessments during treatment. Within-group pretreatment to posttreatment effect sizes (Cohen *d*) were computed for the 4 completers across all 3 measures. Visual inspection of weekly trajectories was used to characterize patterns of symptom change across treatment modules. For the 2 patients who dropped out, we examined their pretreatment scores and available treatment data to explore potential factors contributing to noncompletion.

The interview material is reported descriptively as the patient-perspective component of the case series. No formal qualitative analysis was undertaken. The therapist reviewed their notes from each interview and summarized what each patient said about her experience of the program. These summaries are presented separately for each patient, following the order of the interview topics, without grouping or comparing material across patients. No coding scheme, category system, or qualitative analytic framework was developed or applied; no second researcher analyzed the material independently; and no claim of thematic saturation or of representativeness beyond these 4 patients is made. The material recorded in the therapist’s notes as the patient’s own words is presented in quotation marks; other material is the therapist’s summary of what the patient conveyed and is presented without quotation marks. The quotations were translated from Arabic into English by a bilingual member of the research team.

## Results

### Participant Demographics and Characteristics

Six Arabic-speaking women aged 21 to 50 (mean 34.7, SD 9.8) years were enrolled in the study. All met the criteria for PD, and some had comorbid conditions such as agoraphobia, depression, or generalized anxiety disorder.

### Overview of Findings

The results are presented in 4 parts: treatment engagement and adherence, group-level treatment outcomes, individual patterns of change during treatment, and patients’ perspectives. Individual scores for all 6 patients are presented in Table 1, weekly PDSS-SR scores in Figure 1, and weekly ASI-3 subscale scores in Multimedia Appendix 1.

**Table 1.** Treatment adherence indicators and pretreatment to posttreatment outcomes on the Panic Disorder Severity Scale–Self-Report version (PDSS-SR; clinical threshold ≥7), Anxiety Sensitivity Index-3 (ASI-3; clinical threshold ≥23), and Mobility Inventory (MI; clinical threshold: mean ≥1.5) for the 6 Arabic-speaking patients with panic disorder.^a^

Patient	Modules completed	Completion status	Weeks in treatment	Website logins	PDSS-SR pretreatment	PDSS-SR posttreatment	PDSS 3-month follow-up	ASI-3 pretreatment	ASI-3 posttreatment	MI pretreatment	MI posttreatment
Patient 1	6	Completer	19	39	15	0	0	18	0	1.91	1.18
Patient 2	6	Completer	17	36	11	7	0	29	0	1.44	0
Patient 3	6	Completer	21	32	11	0	7	25	3	1.69	1.07
Patient 4	5	Completer	22	42	18	0	—^b^	59	4	3.82	1.60
Patient 5	1	Noncompleter	10	23	14	14	N/A^c^	38	35	2.34	1.44
Patient 6	4	Noncompleter	19	34	18	22	N/A	59	66	3.81	4.22

a Patients 1‐4 completed the treatment program, whereas patients 5 and 6 dropped out. Higher scores on both measures indicate greater symptom severity.

b Not available; the patient could not be reached for follow-up.

c N/A: not applicable because follow-up assessments were not conducted for the 2 noncompleters.

**Figure 1.**
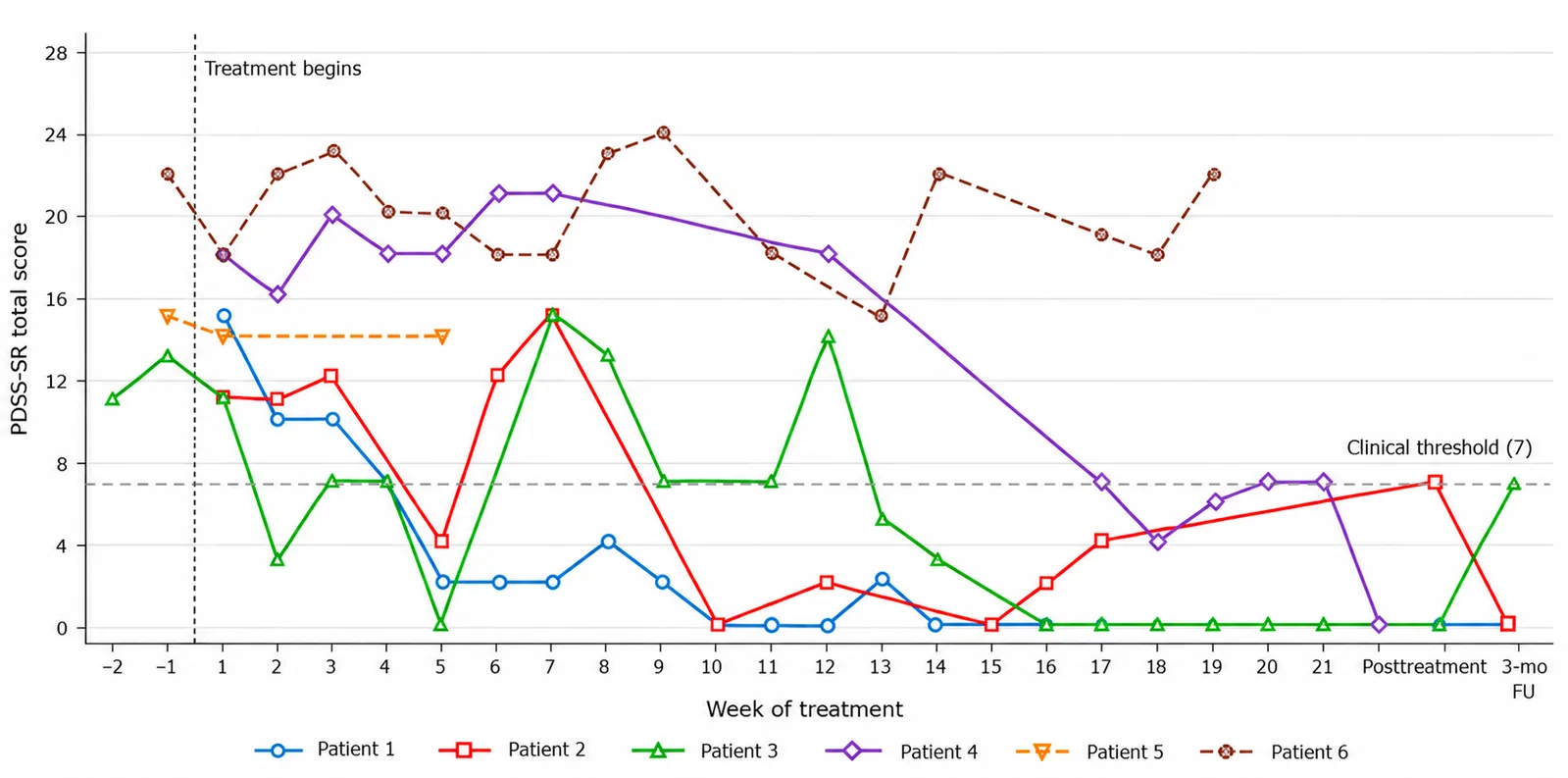
Weekly Panic Disorder Severity Scale–Self-Report (PDSS-SR) total scores for the 6 Arabic-speaking patients with panic disorder. Week 1 is the first week of treatment for every patient. Negative week numbers denote pretreatment assessments completed by the delayed-start group (patients 3, 5, and 6) before treatment began. Posttreatment and follow-up points are shown for patients 1, 2, and 3 only. Patient 4 completed treatment but could not be reached for follow-up; therefore, no posttreatment or follow-up point is plotted for this patient. Patients 5 and 6 discontinued before reaching posttreatment. The dashed horizontal line indicates the PDSS-SR clinical threshold of 7. Gaps in a line indicate weeks for which no assessment was returned. Higher scores indicate greater symptom severity. 3-mo FU: 3-month follow-up.

### Treatment Engagement and Adherence

Table 1 presents engagement indicators and outcome scores for all 6 patients.

The program was completed by 4 of the 6 (67%; patients 1-4) patients. Completers finished within 17 to 22 weeks, logged in to the treatment website 32 to 42 times, and completed most of the assigned worksheets. The 2 noncompleters showed different levels of engagement. Patient 5 dropped out after 10 weeks, having logged in 23 times and completed 1 module; she cited time management difficulties as the main reason. Patient 6 dropped out after 19 weeks, having logged in 34 times and completed 4 of 6 modules, with a 92% worksheet completion rate. During periods of active engagement, she reported reduced avoidance (eg, volunteering, driving in the city, and going out when accompanied), but her engagement fluctuated before she discontinued. Therapist-initiated telephone calls ranged from 1 to 5 per patient, and patients did not always respond to therapist messages.

The therapist’s records indicate that the engagement of patient 4, a completer, was intermittent throughout treatment in the context of child care and household responsibilities, academic assignments, and a period during which her husband and daughter were hospitalized. For patient 6, the records describe family conflict, an unsafe environment, and responsibility for her child as the main obstacles to engagement, alongside frequent and severe panic symptoms.

### Treatment Outcome

Among the 4 completers, scores on all 3 measures decreased from pretreatment to posttreatment. Pretreatment to posttreatment within-group effect sizes, computed for the 4 completers only, were very large: PDSS-SR scores decreased from a mean of 13.75 (SD 3.40) to 1.75 (SD 3.50; Cohen *d*=3.48), ASI-3 scores from 32.75 (SD 18.08) to 1.75 (SD 2.06; *d*=2.41), and MI scores from 2.22 (SD 1.09) to 0.96 (SD 0.68; *d*=1.38). Given the small sample, these effect sizes are presented descriptively. At posttreatment, all 4 completers scored below the ASI-3 threshold (≥23), 3 scored below the PDSS-SR threshold (≥7; patient 2 scored 7), and 3 scored below the MI threshold (mean ≥1.5; patient 4 scored 1.60). Of the 2 noncompleters, patient 6 showed a modest worsening on all 3 measures at the time of discontinuation, and patient 5 showed little change on the PDSS-SR and ASI-3; her lower MI score is difficult to interpret because she completed only 1 module and returned weekly assessments inconsistently.

The 3-month follow-up assessment was completed by 3 of the 4 completers. Patients 1 and 2 had PDSS-SR scores of 0 at follow-up. Patient 3 showed a partial return of symptoms: her PDSS-SR score increased from 0 at posttreatment to 7 at follow-up, and her ASI-3 score increased from 3 to 9. She also described infrequent, partial panic attacks that she was able to manage using skills learned in the program. After the posttreatment assessment, patient 4 notified the therapist that her panic attacks had returned. She did not respond to subsequent contact, so no follow-up scores are available, and this recurrence is reported as a clinical observation.

### Individual Patterns of Symptom Reduction

Weekly PDSS-SR scores for all 6 patients are shown in Figure 1. Patterns of change varied among the completers. Two completers improved steadily, with most change occurring during the cognitive restructuring and exposure modules. One completer showed more fluctuation, with increases in symptoms often coinciding with her menstrual cycle or other life stressors. The fourth completer improved throughout treatment. Weekly MI scores declined for all 4 completers, with the largest decreases generally coinciding with the interoceptive and in vivo exposure modules. Among the noncompleters, patient 6’s avoidance remained high throughout treatment.

### Patients’ Perspectives

Posttreatment interviews were conducted with 4 of the 6 patients (patients 1, 2, 3, and 5). What each patient said is reported below, separately for each patient, following the order of the interview topics. Patient numbers correspond to those in Table 1.

Patient 1 (completer) described the program positively and said it had met her expectations. She said that “the first modules helped me the most, especially psychoeducation concerning fear and panic attacks, specifically the fact that symptoms are not dangerous and the metaphors about my ability to control my PD,” and found the final summary module least useful because it added little that was new. She described the therapeutic relationship as important, and the relationship via the platform as sufficient and efficient; she said she received support whenever she needed it while experiencing the contact as more practical and formal than face-to-face contact. She asked for “the opportunity to record voice messages since writing needs more effort, and at times it made it harder to convey my message precisely.”

Patient 2 (completer) also said the program had met her expectations. She reported that she was not anxious about forgetting material because she could return to it at any time, and that moving between modules helped her see how they fit together. She found the in vivo exposures the most helpful component and the interoceptive exposures less so and suggested that the 2 exposure modules would work better combined. She described the therapeutic relationship as important and the platform’s messaging system as adequate and said that nonverbal communication was the 1 element she missed, as facial expressions and body gestures were sometimes needed to convey a message accurately. She reported procrastination and difficulty establishing regular working times, since she works better with fixed deadlines. She proposed adding several Zoom meetings at points during the program.

Patient 3 (completer) likewise described the program positively. She identified the in vivo exposure module as the most helpful, described the psychoeducation and cognitive restructuring modules as worth revisiting during treatment, and found the interoceptive exposure module the most demanding. She described the therapeutic relationship as important and the relationship via the platform as sufficient for treatment, but said she believed a face-to-face meeting would produce a stronger therapeutic relationship because of nonverbal communication and the greater frequency of contact. During treatment, she once asked the therapist to telephone her because she found a written explanation hard to follow. She reported difficulty finding time for the program and said she dealt with this by working early in the morning or after her children were asleep. She said she “would like to have the opportunity to contact other patients so we can encourage each other and get validation for our experiences.”

Patient 5 (a noncompleter, interviewed after she withdrew) said the program had met her expectations. She described the therapeutic relationship as important and the platform’s messaging system as adequate. She said she could not sustain the program alongside her other commitments: she began treatment during Ramadan, when she needed to focus on fasting, prayer, and worship, and at the end of a school semester in her work as a teacher. She also reported feeling burdened by the weekly questionnaires. Asked what might have enabled her to continue, she answered: “maybe more practical exercises from the beginning, more Zoom meetings with the therapist, and the possibility to share experiences with other participants in order to get validation and motivation.”

## Discussion

### Summary of Findings

This case series had 2 aims. Regarding the first aim, 4 of 6 (67%) patients completed the treatment program. All 4 completers showed reductions on the PDSS-SR, ASI-3, and MI from pretreatment to posttreatment; of the 3 who were assessed at the 3-month follow-up, 2 maintained their gains. Individual patterns of change varied across patients, with the largest reductions generally coinciding with the exposure-focused modules. Regarding the second aim, the 4 interviewed patients spoke positively about the program. Because these interviews were not formally analyzed and included only 4 of the 6 patients, they are reported descriptively.

### Interpretation and Comparison With Existing Literature

The completion rate of 67% (4/6 patients) in our study is comparable to that reported by Strauss et al [13], suggesting similar engagement levels across different cultural contexts. Completers demonstrated high engagement, logging into the treatment website frequently and showing high worksheet completion rates. However, 2 patients dropped out for different reasons. One left due to difficulties managing their time, while the other struggled with severe PD alongside complex life circumstances and interpersonal issues. Both patients also experienced considerable interpersonal issues, raising the question of whether integrating an additional treatment approach, such as psychodynamic or interpersonal therapy, might have been more appropriate for them.

Although the treatment was originally planned for 16 weeks, completers required 17 to 22 weeks to finish the program. This suggests that the original time frame may have been too short for some patients to comfortably complete all 6 modules while managing other life circumstances. Greater scheduling flexibility appeared to support engagement and completion by allowing patients to progress at a pace that better fit their needs. Future implementations may benefit from setting a slightly longer planned treatment window (eg, 18‐22 wk) or building in greater flexibility from the outset rather than treating extensions as exceptions.

Our hypothesis was supported descriptively, with all 4 completers showing reductions in PD symptoms and AS from pretreatment to posttreatment. The maintenance of these improvements at the 3-month follow-up for 2 of the 3 patients assessed is encouraging, suggesting the potential long-term efficacy of ICBT for PD. The greater symptom improvement observed during exposure-focused modules is broadly consistent with CBT models emphasizing exposure as a key mechanism in panic treatment [24]. The varied patterns of improvement observed among patients suggest that personalized treatment approaches may be valuable. While some patients showed steady improvement, others experienced fluctuations related to external factors such as menstrual cycles or life stressors. This variability suggests that future ICBT programs might benefit from built-in flexibility to address individual differences and life circumstances that may impact treatment progress.

The ASI-3 scores observed in this sample can be compared with those reported by Strauss et al [13] for Hebrew-speaking patients who underwent the same ICBT program. Both samples improved on measures of AS and panic symptoms during treatment, and the 2 were broadly comparable at the start of treatment. When individual cases are considered, no consistent pattern emerges: variability appeared more closely tied to differences between individual patients than to language or cultural background, and, with 4 completers and no statistical comparison, we do not interpret any group-level pattern as a cultural difference. This finding differs from Wagner et al [18], who reported that among patients with PD in Australia, those of Arabic background scored higher on fear of dying (physical concerns), whereas those of Australian background scored higher on social concerns such as sweating. This discrepancy may reflect sample size, since larger studies average across patients and may obscure individual differences, whereas our study examined fewer patients in greater depth.

### Limitations

The current study has several limitations. The small sample size, consisting solely of women, limits the generalizability of the findings. Additionally, the reliance on self-reported weekly questionnaires, which were inconsistently completed, requires careful interpretation of the results. Another limitation is the specific inclusion criteria, particularly the decision to include only patients for whom PD was the primary diagnosis. Many PD cases are comorbid with more severe conditions, and excluding such cases may have reduced the generalizability of the results. A further limitation concerns the interview material. The interviews were conducted by the treating therapist, who also wrote the notes on which the account is based and prepared the summaries reported herein. This dual role may have introduced interviewer effects and social desirability bias, in that patients may have been reluctant to voice dissatisfaction directly to the therapist who had treated them, which may have shaped what was recorded and emphasized. The interviews were not audio-recorded, so quotations are as documented in the therapist’s contemporaneous notes rather than verbatim transcriptions; no formal qualitative analysis was undertaken, and no second researcher examined the material independently. Moreover, interviews were conducted with only 4 of the 6 patients, and the 2 who were not interviewed are those whose engagement was most disrupted; therefore, this account is likely to underrepresent the experience of patients who fared least well. Consequently, the interview material should be viewed as an illustrative patient perspective rather than as a systematic analysis of patient experience.

### Future Directions

Future research should address these limitations by replicating this study with a larger, more diverse sample, including both sexes, comorbid conditions, and varying levels of PD severity. This would allow for a more thorough examination of cultural differences in PD and AS following treatment. Additionally, given the inconsistent completion of weekly questionnaires and the difficulty of maintaining regular contact with several patients during treatment, incorporating brief scheduled Zoom meetings could help track patient progress more closely, strengthen therapeutic engagement, and reduce missing data. Finally, some interviewed patients mentioned possible changes to the program, such as voice messages, occasional video meetings, and contact with other patients. Because these comments came from 4 patients and were not formally analyzed, they are noted only as ideas that future studies could examine.

### Conclusions

This case series provides preliminary evidence that an Arabic-translated guided ICBT program for PD was feasible for 6 Arabic-speaking women in Israel. The program was completed by 4 of the 6 patients, and all 4 showed reductions in panic symptoms, AS, and agoraphobic avoidance; 2 of the 3 assessed at the 3-month follow-up maintained their gains. Given the small, all-female sample, the absence of a control condition, and incomplete follow-up, these findings do not establish efficacy or generalizability, and larger controlled studies with Arabic-speaking patients are needed.

## Supplementary material

10.2196/83415 Multimedia Appendix 1 Weekly Anxiety Sensitivity Index-3 (ASI-3) subscale scores for 6 Arabic-speaking patients with panic disorder.

## Abbreviations

AS: anxiety sensitivity
ASI-3: Anxiety Sensitivity Index-3
CBT: cognitive behavioral therapy
DIAMOND: Diagnostic Interview for Anxiety, Mood, and Obsessive-Compulsive and Related Neuropsychiatric Disorders
*DSM-5*: *Diagnostic and Statistical Manual of Mental Disorders, Fifth Edition*
ICBT: internet-based cognitive behavioral therapy
MI: Mobility Inventory
PD: panic disorder
PDSS-IE: Panic Disorder Severity Scale–Interviewer Edition
PDSS-SR: Panic Disorder Severity Scale–Self-Report
