# Supplementary material for: Internet-Based Cognitive Behavioral Therapy for Panic Disorder Among Arabic-Speaking Patients: Case Series
Source: JMIR Form Res. 2026 Nov 12;10:e83415. doi: 10.2196/83415 (PMC13759180; doi:10.2196/83415)
Supplement: Supplementary file 1 [file formative-v10-e83415-s001.docx]

**Multimedia Appendix 2**

*Weekly Anxiety Sensitivity Index-3 (ASI-3) subscale scores for the six Arabic-speaking patients with panic disorder*

**Physical subscale**

| **Assessment** | **P1** | **P2** | **P3** | **P4** | **P5** | **P6** | **M** |
| --- | --- | --- | --- | --- | --- | --- | --- |
| Week 1 | 18 | 13 | 9 | 23 | 8 | 24 | 15.8 |
| Week 2 | 15 | 10 | 11 | 24 | 11 | 24 | 15.8 |
| Week 3 | 9 | 13 | 8 | 24 | — | 24 | 15.6 |
| Week 4 | 4 | 5 | 3 | 24 | — | 24 | 12.0 |
| Week 5 | 3 | 4 | 7 | 24 | — | 24 | 12.4 |
| Week 6 | 3 | 3 | 9 | 24 | — | 23 | 12.4 |
| Week 7 | 3 | 0 | 7 | 24 | — | 24 | 11.6 |
| Week 8 | 0 | 0 | 11 | 23 | — | 24 | 11.6 |
| Week 9 | 0 | 0 | 9 | 8 | — | 24 | 8.2 |
| Week 10 | 0 | 0 | 7 | 9 | — | 24 | 8.0 |
| Week 11 | 1 | 0 | 6 | 12 | — | 24 | 8.6 |
| Week 12 | 0 | 0 | 7 | 11 | — | 22 | 8.0 |
| Week 13 | 0 | 0 | 5 | 10 | — | 23 | 7.6 |
| Week 14 | 0 | 0 | 1 | 4 | — | 23 | 5.6 |
| Week 15 | 0 | — | 3 | — | — | 24 | 9.0 |
| Week 16 | 1 | — | 1 | — | — | 24 | 8.7 |
| Week 17 | 0 | — | 1 | — | — | — | 0.5 |
| Week 18 | 0 | — | 2 | — | — | — | 1.0 |
| Week 19 | 0 | — | 2 | — | — | — | 1.0 |
| Week 20 | 0 | — | 2 | — | — | — | 1.0 |
| 3-mo FU | 1 | 0 | — | — | — | — | 0.5 |

**Cognitive subscale**

| **Assessment** | **P1** | **P2** | **P3** | **P4** | **P5** | **P6** | **M** |
| --- | --- | --- | --- | --- | --- | --- | --- |
| Week 1 | 0 | 10 | 4 | 18 | 17 | 18 | 11.2 |
| Week 2 | 0 | 10 | 7 | 17 | 14 | 24 | 12.0 |
| Week 3 | 0 | 9 | 5 | 18 | — | 23 | 11.0 |
| Week 4 | 1 | 2 | 4 | 18 | — | 24 | 9.8 |
| Week 5 | 0 | 8 | 7 | 21 | — | 22 | 11.6 |
| Week 6 | 0 | 5 | 7 | 21 | — | 22 | 11.0 |
| Week 7 | 0 | 0 | 6 | 15 | — | 23 | 8.8 |
| Week 8 | 0 | 0 | 8 | 15 | — | 19 | 8.4 |
| Week 9 | 0 | 0 | 6 | 6 | — | 24 | 7.2 |
| Week 10 | 0 | 0 | 3 | 0 | — | 15 | 3.6 |
| Week 11 | 0 | 0 | 3 | 3 | — | 19 | 5.0 |
| Week 12 | 0 | 0 | 3 | 0 | — | 21 | 4.8 |
| Week 13 | 0 | 0 | 1 | 0 | — | 23 | 4.8 |
| Week 14 | 0 | 0 | 0 | 0 | — | 21 | 4.2 |
| Week 15 | 0 | — | 3 | — | — | 23 | 8.7 |
| Week 16 | 0 | — | 0 | — | — | 22 | 7.3 |
| Week 17 | 0 | — | 0 | — | — | — | 0.0 |
| Week 18 | 0 | — | 0 | — | — | — | 0.0 |
| Week 19 | 0 | — | 0 | — | — | — | 0.0 |
| Week 20 | 0 | — | 0 | — | — | — | 0.0 |
| 3-mo FU | 0 | 0 | — | — | — | — | 0.0 |

**Social subscale**

| **Assessment** | **P1** | **P2** | **P3** | **P4** | **P5** | **P6** | **M** |
| --- | --- | --- | --- | --- | --- | --- | --- |
| Week 1 | 0 | 6 | 12 | 18 | 13 | 17 | 11.0 |
| Week 2 | 3 | 3 | 11 | 13 | 10 | 24 | 10.7 |
| Week 3 | 2 | 4 | 8 | 14 | — | 20 | 9.6 |
| Week 4 | 1 | 0 | 6 | 13 | — | 24 | 8.8 |
| Week 5 | 1 | 4 | 9 | 14 | — | 23 | 10.2 |
| Week 6 | 1 | 6 | 8 | 12 | — | 18 | 9.0 |
| Week 7 | 1 | 0 | 8 | 13 | — | 23 | 9.0 |
| Week 8 | 0 | 0 | 11 | 8 | — | 20 | 7.8 |
| Week 9 | 0 | 0 | 12 | 6 | — | 24 | 8.4 |
| Week 10 | 0 | 0 | 12 | 1 | — | 18 | 6.2 |
| Week 11 | 0 | 0 | 9 | 1 | — | 20 | 6.0 |
| Week 12 | 0 | 0 | 9 | 0 | — | 20 | 5.8 |
| Week 13 | 0 | 0 | 8 | 0 | — | 23 | 6.2 |
| Week 14 | 0 | 0 | 2 | 0 | — | 20 | 4.4 |
| Week 15 | 0 | — | 6 | — | — | 23 | 9.7 |
| Week 16 | 0 | — | 0 | — | — | 20 | 6.7 |
| Week 17 | 0 | — | 1 | — | — | — | 0.5 |
| Week 18 | 0 | — | 2 | — | — | — | 1.0 |
| Week 19 | 0 | — | 2 | — | — | — | 1.0 |
| Week 20 | 0 | — | 1 | — | — | — | 0.5 |
| 3-mo FU | 0 | 0 | — | — | — | — | 0.0 |

*Note*. P1–P6 = Patients 1–6, numbered as in Table 1. Week 1 represents each patient’s pre-treatment assessment, and subsequent rows represent weekly assessments during treatment. Each ASI-3 subscale comprises six items rated from 0 to 4, yielding a possible score of 0–24; higher scores indicate greater anxiety sensitivity. The dash (—) indicates weeks for which no assessment was available, either because the patient did not complete the weekly questionnaire or because she had completed or discontinued treatment. M = mean of the patients who provided data for that week. Patients 5 and 6 discontinued treatment; Patient 4 completed treatment but could not be reached for follow-up. 3-mo FU = 3-month follow-up assessment, available for Patients 1 and 2. Week 1 scores and each patient's final treatment-week scores correspond to the pre- and post-treatment ASI-3 scores reported in Table 1.
